# COSMICA: A Novel Dataset for Astronomical Object Detection with Evaluation Across Diverse Detection Architectures

**DOI:** 10.3390/jimaging11060184

**Published:** 2025-06-04

**Authors:** Evgenii Piratinskii, Irina Rabaev

**Affiliations:** Software Engineering Department, Shamoon College of Engineering, 56 Bialik St., Be’er Sheva 8410802, Israel; irinar@ac.sce.ac.il

**Keywords:** astronomical object detection, deep learning, YOLO models, telescope image analysis, astronomy dataset

## Abstract

Accurate and efficient detection of celestial objects in telescope imagery is a fundamental challenge in both professional and amateur astronomy. Traditional methods often struggle with noise, varying brightness, and object morphology. This paper introduces COSMICA, a novel, curated dataset of manually annotated astronomical images collected from amateur observations. COSMICA enables the development and evaluation of real-time object detection systems intended for practical deployment in observational pipelines. We investigate three modern YOLO architectures, YOLOv8, YOLOv9, and YOLOv11, and two additional object detection models, EfficientDet-Lite0 and MobileNetV3-FasterRCNN-FPN, to assess their performance in detecting comets, galaxies, nebulae, and globular clusters. All models are evaluated using consistent experimental conditions across multiple metrics, including mAP, precision, recall, and inference speed. YOLOv11 demonstrated the highest overall accuracy and computational efficiency, making it a promising candidate for real-world astronomical applications. These results support the feasibility of integrating deep learning-based detection systems into observational astronomy workflows and highlight the importance of domain-specific datasets for training robust AI models.

## 1. Introduction

Automated detection of celestial objects in astronomical images is a growing need in both professional and amateur observational astronomy. As large-scale sky surveys and hobbyist astrophotography become increasingly common, the ability to rapidly identify and localize objects such as galaxies, comets, and nebulae is essential for real-time observation, data analysis, and educational use. Manual analysis remains time-consuming and subjective, while traditional image processing techniques are limited in handling faint objects, noise, and varying imaging conditions.

Recent advances in deep learning, particularly convolutional neural networks (CNNs), have demonstrated strong potential for object detection tasks. Among these, the YOLO (You Only Look Once) family of architectures has gained wide adoption due to its speed and efficiency, making it suitable for deployment in constrained environments such as portable telescopes or embedded systems.

This work introduces the COSMICA dataset—a large annotated collection of astrophotographic images tailored for training and evaluating object detection models—and investigates the performance of three recent YOLO variants and two alternative architectures. The goal is to assess which architecture offers the best trade-off between accuracy, speed, complexity, and deployability in the context of practical astronomical object detection. All the models are trained and evaluated on the COSMICA dataset, which consists of 5145 manually annotated telescope images, sourced from the astronomy.ru (accessed on 4 May 2025) community, the largest Russian language forum for amateur astronomy enthusiasts. On the forum, hobbyists share their astrophotographic works, which are organized by object category—an arrangement that greatly facilitated the dataset compilation process.

- **Astronomical object detection dataset.** Compilation of COSMICA—a large, well-annotated dataset featuring telescope images manually labeled into four categories: comets, galaxies, nebulae, and globular clusters. The dataset has been made publicly available on Kaggle [1] and Roboflow [2].
- **Model comparison.** A systematic evaluation and comparison of YOLOv8 [3], YOLOv9 [3], YOLOv11 [4], EfficientDet-Lite0 [5], and MobileNetV3-FasterRCNN-FPN [6] for astronomical object detection using a consistent experimental pipeline.

The remainder of this paper is structured as follows: Section 2 provides an overview of previous research in astronomical object detection and the application of deep learning-based approaches in the field. Section 3 describes the preparation of the dataset, including data collection, annotation, and preprocessing techniques. Section 4 presents an overview of the deep learning architectures used in this study. Section 5 details the training pipeline and hyperparameter configurations. Section 6 discusses the experimental results and provides an analysis of model performance, while Section 7 outlines the limitations and challenges encountered in this study. Finally, conclusions and potential future research directions are presented in Section 8.

## 2. Related Work

In recent years, deep learning has increasingly become a cornerstone for astronomical object detection, largely driven by the growing availability of large-scale sky surveys and high-resolution imaging data. The shift from traditional computer vision techniques to deep neural networks reflects the increasing complexity and volume of astronomical datasets. Below, we provide an overview of recent advances in the field.

A notable trend has been the adoption of specialized neural network architectures tailored for classification and detection tasks in astronomy. Zhang et al. [7] proposed HR-CelestialNet, a deep learning-based classification model specifically designed to identify galaxies and nebula/star clusters in images captured by the Hubble Space Telescope. Their findings showed that deep neural networks can outperform conventional models such as AlexNet, VGGNet, and ResNet in terms of both accuracy and processing speed, while also maintaining robustness in low-quality or partially occluded image scenarios. Another key direction of research is the application of pattern recognition and shape analysis techniques to astronomical imagery. Khlamov et al. [8] introduced analytical models and pattern recognition approaches to classify a variety of astronomical object shapes, including circular, elongated, blurred, and intersecting forms. Their framework emphasizes the value of shape-based analysis for enhancing classification reliability, especially when dealing with faint or ambiguous objects that are commonly encountered in observational data. In parallel, more comprehensive studies have explored the general applicability of AI techniques across various astronomical tasks. Lee et al. [9] provided an extensive review of modern AI-driven approaches for celestial object detection and anomaly identification. Their work demonstrated that neural networks, combined with advanced preprocessing techniques, can significantly accelerate the analysis of large-scale datasets while enhancing detection sensitivity.

Recent years have also seen increased efforts to adapt and evaluate deep learning models directly within the context of astronomical image processing. For instance, Shankar et al. [10] investigated the use of VGG16, VGG19, and MobileNetV2 for classifying galaxy morphologies, demonstrating superior accuracy and robustness compared with classical CNNs. Ramya et al. [11] applied Recursive Convolutional Neural Networks (RCNNs) to a large-scale image dataset containing diverse celestial objects, achieving over 93% classification accuracy. Parisot and Jaziri [12] introduced a YOLO-based deep learning pipeline for Electronically Assisted Astronomy (EAA), enabling real-time detection and enhancement of deep sky objects captured through smart telescopes. In a broader benchmarking context, Cornudet et al. [13] presented DeepDISC, a comparative framework evaluating several object detection architectures for astronomy, highlighting the advantages of Transformer-based models and domain-specific tuning. Parisot [14] introduced the DeepSpaceYoloDataset, comprising annotated images captured via smart telescopes, which facilitates targeted training of deep learning models for real-world astrophotography. These studies collectively underscore the importance of customized neural network designs and astronomy-focused training pipelines in achieving reliable object detection across varied observational environments.

Although not directly related to astronomical imaging, recent studies in other domains have shown the effectiveness of deep learning models in challenging detection environments. For instance, Tan et al. [15] proposed a segmentation-based neural network for foreign object detection in urban rail systems operating under noisy and dynamic conditions. Similarly, Tang et al. [16] introduced a hybrid CNN–Transformer model for micro-expression recognition, achieving high accuracy in identifying subtle features. These works highlight the broader applicability of deep learning architectures to object detection tasks involving low contrast and high noise challenges that are also prevalent in astronomical imaging.

While previous studies have often focused on individual model versions or specific object categories, few have conducted comprehensive comparative evaluations of state-of-the-art deep learning architectures for multi-class astronomical object detection. Our study addresses this gap by systematically analyzing YOLOv8, YOLOv9, YOLOv11, EfficientDet-Lite0, and MobileNetV3-FasterRCNN-FPN models trained under identical conditions. Moreover, we assess not only their detection accuracy but also their computational efficiency, which is essential for real-time and resource-constrained applications in astronomy.

## 3. Dataset Preparation

As part of this study, we assembled a large-scale dataset named *COSMICA* (COmets, Star-clusters, Messier Images Captured by Amateurs). The dataset comprises telescope images gathered from the astronomy.ru (accessed on 4 May 2025) community, a Russian online forum where amateur and professional astronomers regularly share astrophotographic observations. *COSMICA* was compiled using a custom-developed web-scraping script to automatically retrieve images from a designated forum thread. Subsequently, each downloaded image was manually reviewed to eliminate irrelevant, duplicated, or low-quality content, ensuring that the dataset consisted exclusively of high-quality images suitable for annotation and training of astronomical object detection models.

### 3.1. Data Annotation

The dataset was semi-manually annotated using the Roboflow platform [17]. In the beginning, we selected sampled images and manually labeled each image with bounding boxes corresponding to four primary categories of celestial objects: comets, galaxies, nebulae, and globular clusters. After the initial manual annotation, an experimental set of trained models was employed to assist in the annotation process. These models generated preliminary bounding boxes, which were then manually reviewed and corrected by the author. Incorrect predictions were adjusted, missing objects were added, and unnecessary detections were removed to ensure high-quality annotations. The final dataset consists of 5145 unique images, each potentially containing multiple astronomical objects. As a result, the dataset includes 8859 annotated object instances across the dataset, as shown in Table 1.

It is important to note that the number of images per class in the COSMICA dataset is not uniformly distributed. This imbalance stems from the nature of the source data, which were collected from astronomy forums where amateur and professional astronomers share their observations. Certain celestial objects, such as galaxies, are not only more prevalent in the observable universe but also more visually striking and easier to capture with amateur equipment. As a result, they are more frequently photographed and shared. In contrast, objects like globular clusters are comparatively rarer in user-generated astrophotography, both due to their limited number and the higher observational difficulty. Therefore, the dataset reflects a natural distribution based on the observational preferences and practical constraints of the contributing community.

**Figure 1:**
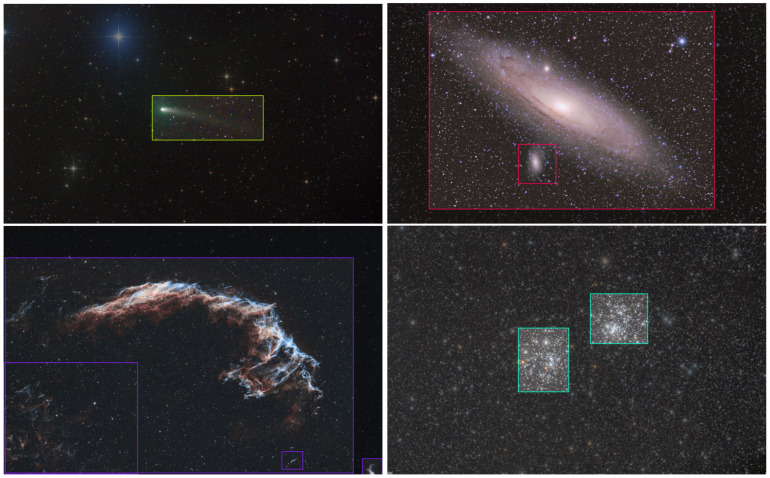
Examples of annotated images from the dataset: red: galaxies, blue: nebulae, yellow: comets, and green: globular clusters.

To illustrate the annotation quality and diversity of astronomical objects in the dataset, Figure 1 presents representative examples of labeled images from each class. These examples highlight how the annotation process ensures accurate bounding box placements, even for complex and overlapping celestial structures.

### 3.2. Preprocessing and Augmentation

The original images, collected from various posts on the astronomy.ru forum, showed considerable variation in format and quality—some were in grayscale, while others were in color, and their dimensions varied widely. To standardize the dataset, all images were resized to a fixed resolution of 640×640 pixels using zero padding to preserve the aspect ratio. This resolution was empirically chosen as it strikes a balance between retaining detail for small celestial objects and maintaining computational efficiency for training. Additionally, all images were converted to grayscale to unify the input format and emphasize structural and morphological features relevant to object detection.

To enhance model generalization and artificially enlarge the dataset, a combination of augmentations was applied to each image during preprocessing in Roboflow [17]. For every original training sample, seven augmented variants were generated, with each version incorporating a randomized mix of transformations, such as flipping (horizontal and vertical), rotation (up to ±45°), brightness (±25%) and exposure (±15%) adjustment, slight Gaussian blur (up to 1.6 pixels), and random noise (affecting up to 1.68% of pixels). This approach allows the model to learn from a broader range of visual variations, improving robustness to real-world observational noise and inconsistencies.

### 3.3. Dataset Split

To ensure an even class ratio distribution across training, validation, and testing sets, a custom script was developed. The final distribution of images across the three sets is summarized in Table 2. This split corresponds approximately to a 79/11/11 ratio for training, validation, and testing sets, respectively.

**Figure 2:**
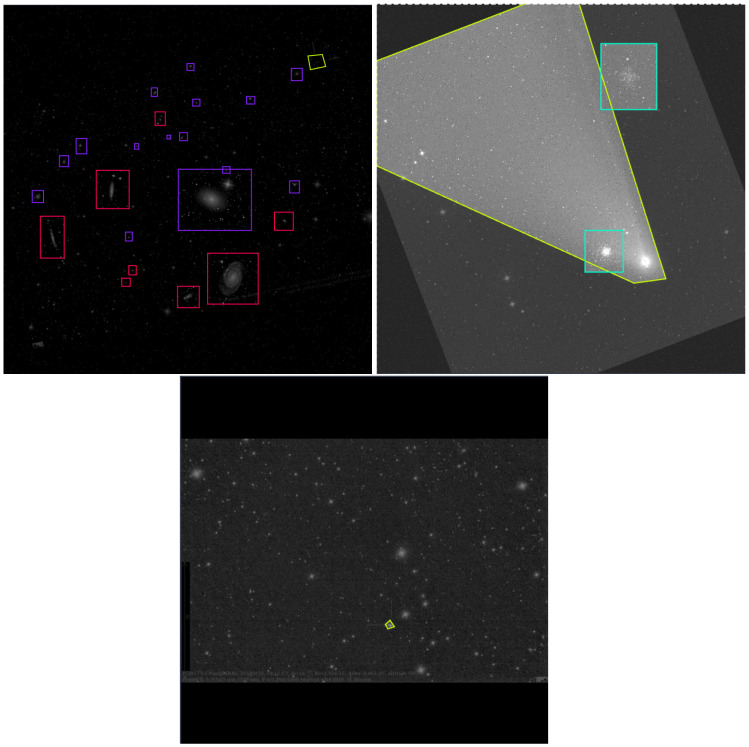
Examples of annotated images from the dataset after preprocessing: red: galaxies, blue: nebulae, yellow: comets, and green: globular clusters.

Figure 2 presents examples of images from the *COSMICA* dataset after preprocessing, showcasing bounding box annotations for different celestial object classes. The annotations were initially created on the original images prior to preprocessing and were subsequently transformed to remain consistent with the augmented and resized data. To further improve object–background separation, an additional set of background-only images was generated and added to the *COSMICA* dataset. Details of this additional augmentation process are provided in Section 6.6. The complete *COSMICA* dataset, including all annotations, has been made publicly available on both Kaggle [1] and Roboflow [2] platforms.

## 4. Model Architectures

In this study, we experimented with three YOLO-based object detection models—YOLOv8 [3], YOLOv9 [18], and YOLOv11 [4]. Each of these models follows the general YOLO paradigm, which enables real-time object detection by processing an image in a single forward pass through a convolutional neural network. However, these variants differ in their architectural complexity, parameter count, and computational efficiency. In addition to YOLO-based models, we explored two alternative architectures, EfficientDet-Lite0 [5] and MobileNetV3-FasterRCNN-FPN [6], to broaden the scope of the comparison and offer further perspective on the behavior of different detection paradigms when applied to astronomical imagery.

### 4.1. YOLO-Based Models

The selection of YOLO-based models for this study was motivated by their suitability for real-world deployment in field conditions. Due to their fast inference speed and relatively low computational overhead, YOLO models can be efficiently executed on a wide range of hardware, including portable devices such as Raspberry Pi or NVIDIA Jetson boards connected to astrocameras. This makes it feasible to integrate object detection directly into observational equipment, enabling real-time identification of celestial objects during night-sky sessions. Furthermore, the YOLO architecture offers excellent cross-platform compatibility, with support for deployment on desktop, mobile, and embedded systems via export to formats such as ONNX, CoreML, and TensorRT. These characteristics make YOLO a particularly practical and versatile solution for astronomical object detection, aligning well with the needs of both research and amateur astronomy communities. YOLOv8 [3] was chosen as a widely used and optimized variant with a strong balance between accuracy and performance. YOLOv9 [18] was included due to its enhanced feature extraction capabilities, integrating Transformer-based attention mechanisms that improve object localization. YOLOv11 [4], as one of the latest iterations, was selected for its advanced architectural optimizations, including a hybrid CNN–Transformer backbone, which has the potential to further improve detection performance on complex astronomical images. Additionally, these models were selected based on hardware constraints, ensuring that they could be efficiently trained on an NVIDIA RTX 3060 GPU while still achieving competitive results.

It is worth noting that YOLOv10 was not included in this comparative analysis for several reasons. Unlike YOLOv8, YOLOv9, and YOLOv11, which are all officially maintained and documented within the Ultralytics ecosystem, YOLOv10 was developed by a different research group (YOLO-World) and is not part of the core Ultralytics release cycle. Moreover, YOLOv10 focuses primarily on generalized object detection and zero-shot learning scenarios, making it less suitable for tasks involving detection of known object categories, such as the domain-specific astronomical objects studied in this paper. Additionally, at the time of experimentation, a standardized and well-documented training pipeline for domain-specific fine-tuning of YOLOv10 was not readily available, which limited its practical applicability in this context.

Table 3 presents a comparison of the models in terms of the number of layers, number of parameters, and computational complexity measured in Giga Floating Point Operations (GFLOPs) per inference. While GFLOPs do not reflect hardware requirements directly, they provide an estimate of the amount of computation a model performs during inference, which is important for understanding its runtime efficiency on different devices. In the following sections, we provide an overview of each model architecture.

#### 4.1.1. YOLOv8 Architecture

YOLOv8 [3] is a modern refinement of the original YOLO [19] series, designed with an optimized backbone and neck structure to improve feature extraction and spatial information retention. The architecture incorporates depth-wise convolutional layers, which reduce computational cost while maintaining accuracy. It also utilizes the CSPDarknet backbone [20], which enhances gradient flow and minimizes redundancy in feature maps. To improve multi-scale feature aggregation, YOLOv8 employs a PANet neck [21], facilitating better fusion of spatial and semantic information. Additionally, the model leverages the SIOU loss function [22], which enhances convergence and precision during training.

The YOLOv8 model used in this study consists of 268 layers, with 43.6 million parameters, and requires 164.8 GFLOPs for inference.

#### 4.1.2. YOLOv9 Architecture

YOLOv9 [18] builds upon YOLOv8 by integrating advanced attention mechanisms and a more efficient Feature Pyramid Network, further enhancing object detection capabilities. One of the key improvements is the incorporation of Transformer-based attention layers, which enable better contextual understanding by capturing long-range dependencies in the input data. Additionally, YOLOv9 employs an enhanced Feature Pyramid Network, which improves the detection of objects at different scales by refining multi-level feature fusion. Another notable enhancement is the introduction of dynamic activation functions, which adjust non-linearity based on the complexity of extracted features, leading to improved adaptability and robustness.

The YOLOv9 model used in this study consists of 384 layers with 25.3 million parameters, and requires 102.3 GFLOPs for inference.

#### 4.1.3. YOLOv11 Architecture

YOLOv11 [4] represents the latest iteration in the YOLO family, designed to maximize detection accuracy while maintaining computational feasibility. One of its key advancements is the integration of a hybrid CNN–Transformer backbone combining convolutional and Transformer-based feature extraction to improve object localization and contextual understanding. Additionally, YOLOv11 employs an improved decoupled head, allowing for the separate optimization of localization and classification, leading to enhanced detection performance. Another critical enhancement is the incorporation of adaptive receptive field modules, which dynamically adjust receptive fields to better capture fine-grained features, making the model particularly effective for detecting objects with varying scales and structures.

The YOLOv11 model used in this study consists of 464 layers with 25.2 million parameters, and requires 86.6 GFLOPs for inference.

### 4.2. EfficientDet-Lite0 and MobileNetV3-FasterRCNN-FPN

To extend the comparative evaluation beyond the YOLO family, two widely used non-YOLO object detection models were also examined, EfficientDet-Lite0 [5] and MobileNetV3-FasterRCNN-FPN [6]. These models have been adopted in various real-world applications due to their practical trade-offs between accuracy and computational efficiency.

EfficientDet-Lite0 [5] is based on the EfficientDet family and incorporates a compound scaling method alongside a BiFPN (Bidirectional Feature Pyramid Network), which allows for efficient multi-scale feature fusion. The “Lite” variant is optimized for deployment scenarios requiring fast inference and memory efficiency, particularly on edge devices.

MobileNetV3-FasterRCNN-FPN [6] integrates the MobileNetV3 backbone with the region-based Faster R-CNN detection framework and an FPN for multi-scale object detection. This configuration aims to improve object localization performance while maintaining general compatibility with mainstream detection pipelines.

To contextualize the architectural efficiency of these lightweight models, Table 4 presents a comparison of their structural characteristics in terms of depth (number of layers), parameter count, and GFLOPs.

## 5. Experimental Settings

To ensure a fair comparison, all the models were trained using the same dataset, augmentation strategies, and hyperparameter settings.

### 5.1. Training Configuration

A batch size of 16 was used for all training runs, with a maximum of 400 epochs, applying early stopping if no improvement was observed for 20 consecutive epochs. The training process utilized adaptive learning rate optimization with an initial learning rate of 0.01, a momentum value of 0.937, and a weight decay of 0.0005. The Intersection over Union (IoU) threshold was set to 0.7 as a trade-off between precision and recall.

All models were initialized with pretrained weights from the COCO dataset [23] and trained in mixed-precision mode to optimize computational efficiency. Mixed-precision training leverages both 16-bit (half-precision) and 32-bit (single-precision) floating-point computations, reducing memory usage and accelerating training without significantly compromising model accuracy.

**Figure 3:**
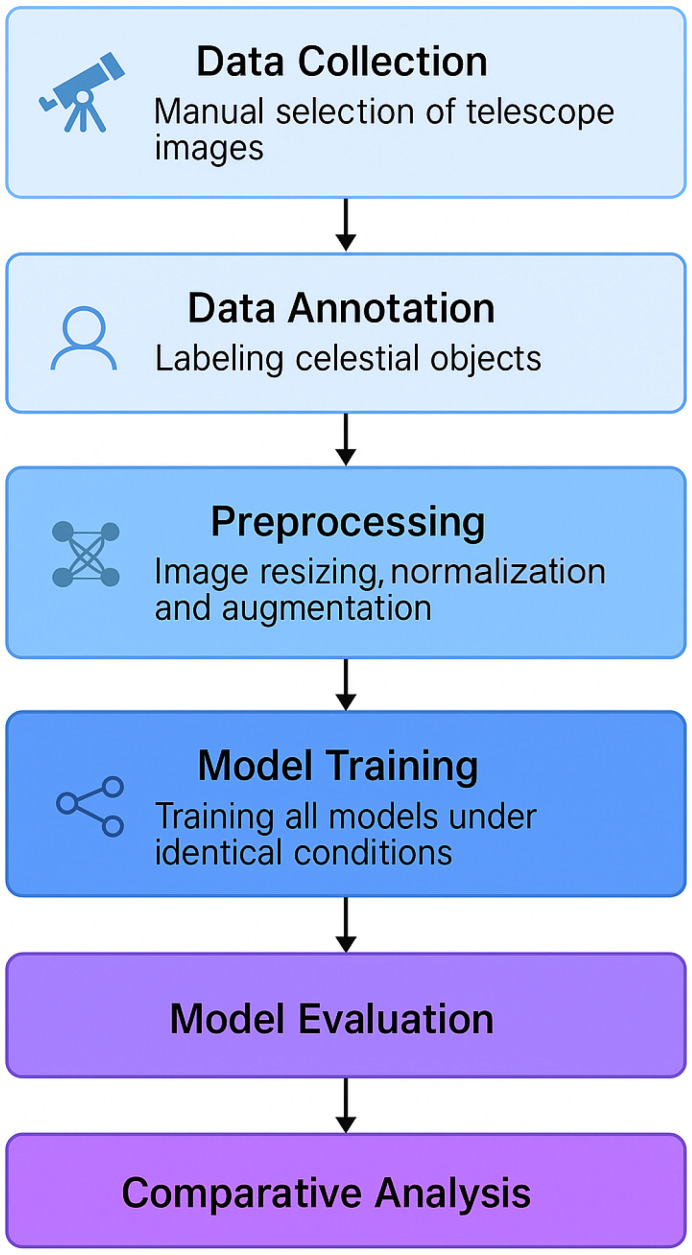
Illustration of the training pipeline employed in this study, outlining the process from data collection and annotation to model training, evaluation, and final comparison.

The training workflow follows a structured pipeline, as illustrated in Figure 3.

1. **Dataset Preparation**: The first step involves preprocessing and augmenting the input images using various transformations to improve model generalization. These transformations, including flipping, rotation, brightness adjustments, and noise injection, are detailed in Section 3.
2. **Model Initialization**: Each YOLO model is initialized with pretrained weights from the COCO dataset and configured for mixed-precision training to optimize memory usage and computational efficiency.
3. **Training Phase**:
  3.1. **Training**: Training was performed using mini-batches of 16 images, and the network parameters are updated iteratively using stochastic gradient descent. During this phase, the model learns feature representations for astronomical object detection.
  3.2. **Validation**: After each training epoch, the model is evaluated on the validation set. The primary performance metric used is mean Average Precision (mAP), which assesses the accuracy of object detection across different categories. The mAP is computed as
    (1) $$\mathrm{mAP}=\frac{1}{N}\sum_{i=1}^{N}\int_{0}^{1}P_{i}(r)\;dr$$
    where *N* is the number of object classes, and $P_{i}(r)$ represents the precision–recall curve for class *i*, integrated over the recall *r* range from 0 to 1.
  3.3. **Early Stopping**: To prevent overfitting, training is halted if no performance improvement is observed for 20 consecutive epochs. This ensures optimal model generalization to unseen data.

All models in this study were initialized using pretrained weights from the COCO dataset, a widely adopted strategy in computer vision that provides a strong baseline for transfer learning. This approach enables faster convergence and reduces the required volume of domain-specific labeled data, which is particularly beneficial in resource-constrained academic settings.

### 5.2. Loss Functions

Each YOLO variant uses a combination of loss functions to optimize detection performance, as follows:

- **Bounding Box Loss** ($L_{\mathrm{box}}$): Regression loss based on CIoU (Complete Intersection over Union).
- **Classification Loss** ($L_{\mathrm{cls}}$): Binary cross-entropy loss for object classification.
- **Distribution Focal Loss** ($L_{\mathrm{dfl}}$): Applied to refine bounding box prediction confidence.

The total loss is computed as

(2) $$L_{\mathrm{total}}=\lambda_{\mathrm{box}}L_{\mathrm{box}}+\lambda_{\mathrm{cls}}L_{\mathrm{cls}}+\lambda_{\mathrm{dfl}}L_{\mathrm{dfl}},$$

where $\lambda_{\mathrm{box}}$, $\lambda_{\mathrm{cls}}$, and $\lambda_{\mathrm{dfl}}$ are weight coefficients tuned to balance different loss components.

### 5.3. Hardware and Computational Efficiency

The experiments were conducted on a machine equipped with an NVIDIA RTX 3060 GPU (12 GB VRAM), an AMD Ryzen 7 5800X CPU, and 32 GB of DDR4 RAM. All models were trained using the PyTorch 2.0 framework.

The total training time per model took approximately 50 h. Table 5 summarizes the hardware and training parameters.

## 6. Results and Discussions

This section presents the evaluation results of the three YOLO models—YOLOv8, YOLOv9, and YOLOv11—on the test set. Performance is assessed based on multiple key metrics, including mean Average Precision, precision, recall, and computational efficiency.

- **Mean Average Precision (mAP@50 and mAP@50–95)**: mAP@50 measures the average precision at a single IoU threshold of 0.5. In contrast, mAP@50–95 calculates the Average Precision (AP) at multiple IoU thresholds ranging from 0.5 to 0.95 (with a step size of 0.05) and then averages these AP values to provide a comprehensive evaluation of model performance across different localization strictness levels.
- **Precision**: The ratio of correctly detected astronomical objects to all positive detections.
- **Recall**: The proportion of true astronomical objects correctly detected by the model.
- **Inference Speed in Frames per Second (FPS)**: The number of frames per second the model processes during inference.
- **Loss Values**: Includes box loss ($L_{\mathrm{box}}$), classification loss ($L_{\mathrm{cls}}$), and distribution focal loss ($L_{\mathrm{dfl}}$), as described in Section 5.2.

### 6.1. YOLO Model Evaluation

Table 6 presents a comparison of YOLOv8, YOLOv9, and YOLOv11 based on the standard evaluation metrics (mAP, precision, recall, FPS) computed on the held-out test set.

As shown in Table 6, YOLOv11 achieves the highest mAP@50–95 (0.6187), precision (0.9082), and recall (0.8193) while also maintaining the best inference speed (18.21 FPS). This superior performance can be attributed to its hybrid CNN–Transformer architecture and the decoupled detection head, which allows it to efficiently extract and process spatial and contextual features while maintaining computational efficiency. YOLOv9, which integrates Transformer-based attention mechanisms and an enhanced Feature Pyramid Network, performs second best and demonstrates a strong balance between accuracy and inference speed. YOLOv8, which has the largest number of parameters due to its heavier backbone and neck design, shows lower inference speed and slightly reduced performance. This suggests that a higher parameter count alone does not guarantee better accuracy and may negatively impact real-time applicability.

### 6.2. Performance of EfficientDet-Lite0 and MobileNetV3-FasterRCNN-FPN

To explore lightweight alternatives to YOLO-based models for deployment in mobile or embedded scenarios, we performed experiments with EfficientDet-Lite0 and MobileNetV3-FasterRCNN-FPN models. The results are presented in Table 7.

EfficientDet-Lite0 achieved a mAP@50 of 0.2312 and a mAP@50:95 of 0.0827, with an AR@100 of 0.1735. MobileNetV3-FRCNN performed slightly better with a mAP@50 of 0.4206, mAP@50:95 of 0.1889, and AR@100 of 0.3379. These comparably low scores reflect the models’ inability to robustly detect faint and morphologically diverse celestial structures present in our dataset. While both models are known for their computational efficiency, the results indicate substantial underperformance compared with YOLO-based models. Error analysis revealed that both models underperformed on the COSMICA dataset, especially in detecting faint or small-scale astronomical objects. In particular, EfficientDet-Lite0 failed to register any meaningful detections of small objects, with mAP_small = 0.0. MobileNetV3-FRCNN showed moderately improved accuracy, yet still struggled with high false-negative rates and background differentiation.

Although EfficientDet-Lite0 and MobileNetV3-FasterRCNN-FPN were initially considered as potential alternatives, their insufficient accuracy in the context of our domain-specific dataset led to their exclusion from subsequent comparative analysis.

### 6.3. Bootstrapped Evaluation and Statistical Analysis

To strengthen the statistical validity of our findings and address reviewer concerns about generalization, we performed a bootstrapped evaluation. Bootstrapping is a resampling technique that estimates the distribution of a statistic (e.g., mean, precision) by drawing samples with replacement from the test set. It enables the estimation of confidence intervals (CIs) without requiring additional data or computationally intensive cross-validation. This method is especially useful when evaluating models on domain-specific datasets like COSMICA.

For each YOLO model, 1000 bootstrap replicates were generated. We employed stratified sampling to ensure that each bootstrap replicate maintained the same class distribution as the original test set, which is particularly important given our imbalanced dataset. On each replicate, we computed four key metrics: mean Average Precision at IoU thresholds of 0.50–0.95 (mAP@50–95), mAP@50, precision, and recall. Table 8 reports the estimated means and their associated 95% confidence intervals.

**Figure 4:**
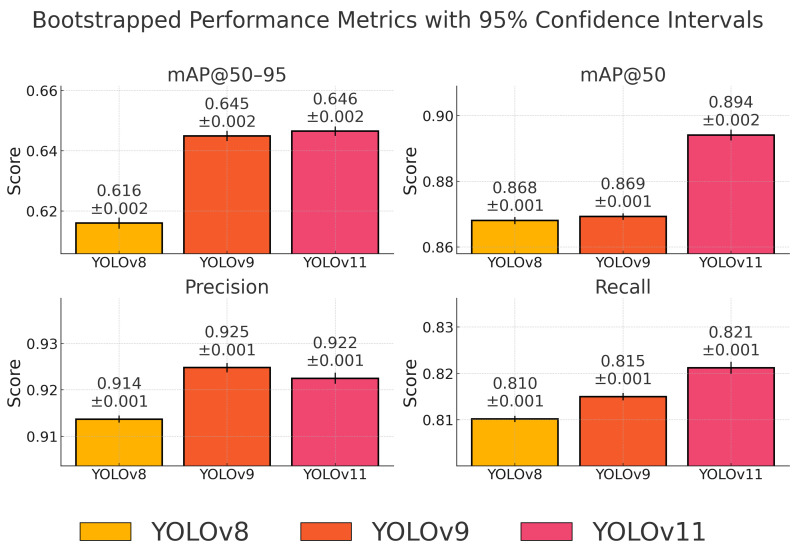
Bootstrapped performance metrics for each YOLO model with 95% confidence intervals. Bars represent mean scores across 1000 resampled test sets.

Figure 4 illustrates the mean performance metrics obtained through bootstrapped evaluation, along with 95% confidence intervals. The visual comparison confirms the quantitative findings: YOLOv11 achieves the best results in terms of recall and mAP@50, while also maintaining a strong balance in precision and overall localization accuracy (mAP@50–95). Notably, the confidence intervals between YOLOv9 and YOLOv11 for precision and mAP@50–95 overlap, indicating no statistically significant difference. This supports our conclusion that YOLOv11 provides the most robust and generalizable performance for astronomical object detection under real-world conditions.

The bootstrapped results confirm that YOLOv11 achieves the highest overall detection performance, particularly in terms of mAP@50–95, mAP@50, and recall. Its precision is statistically indistinguishable from that of YOLOv9, with overlapping confidence intervals. YOLOv9 remains a strong alternative, offering a slightly higher mean precision, while YOLOv8, although efficient, underperforms across all major metrics.

This statistical evaluation is consistent with the previously reported results in Section 6.1 and validates our conclusion that YOLOv11 is the most balanced and generalizable model for astronomical object detection tasks. The combination of high recall and strong localization accuracy makes it especially suitable for use in real-time observational pipelines, where completeness is critical.

### 6.4. Training Convergence

**Figure 5:**
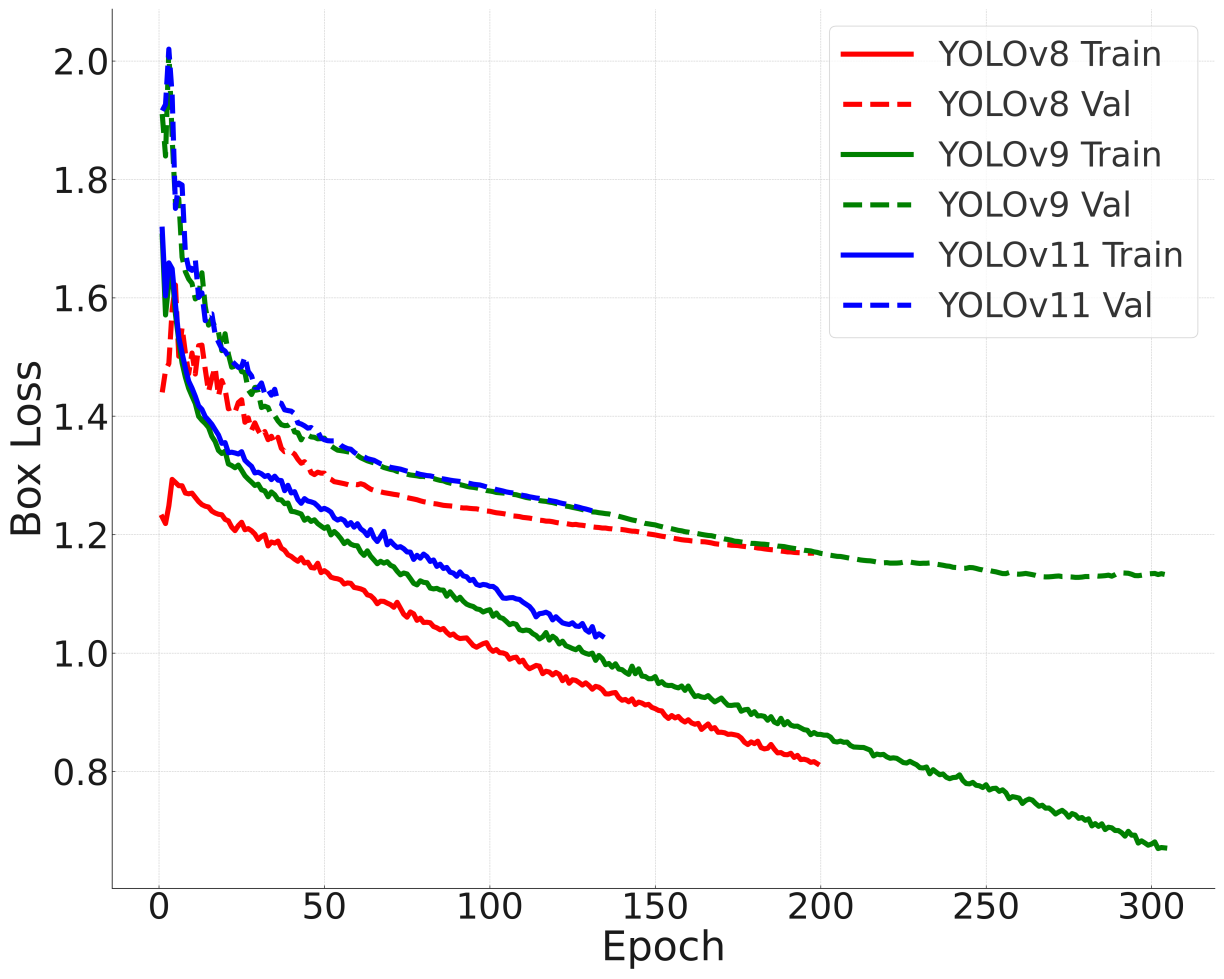
Training loss curves for YOLOv8, YOLOv9, and YOLOv11, with early stopping applied when no improvement was observed.

To analyze training stability, Figure 5 illustrates the loss curves for all three models. Analyzing the convergence behavior provides insights into the optimization dynamics of each model, helping to assess their learning efficiency and potential risks of overfitting or underfitting.

The convergence behavior of the models indicates that YOLOv11 reaches lower final loss values compared with YOLOv8 and YOLOv9. Early stopping was triggered at 199 epochs for YOLOv8, 304 epochs for YOLOv9, and 214 epochs for YOLOv11. This indicates that YOLOv8 reached its optimal performance the earliest, suggesting that it converged quickly but may have had limited capacity for further improvement. In contrast, YOLOv9 required more epochs to converge, reflecting a slower but more gradual optimization process. YOLOv11 demonstrated a favorable balance, achieving the lowest final loss within a moderate number of epochs, which indicates both efficient learning and strong generalization ability.

### 6.5. Per-Class Performance

To provide a detailed analysis of class-wise detection performance, Table 9 and Table 10 present the mAP@50 and mAP@50–95 scores, respectively, for each model and each celestial object class. YOLOv11 achieves the highest per-class mAP values across all four classes in both metrics, demonstrating its superior generalization capabilities.

In addition to the mAP scores, Table 11, Table 12 and Table 13 present normalized confusion matrices for each model, providing deeper insight into their per-class classification behavior. These matrices reveal a common source of error: some astronomical objects are misclassified as background. This issue is most notable for galaxies and nebulae in all three models, where a significant portion of the misclassifications occur along the background column. Such misclassification is especially problematic for faint or diffuse objects, which are often visually similar to noisy background regions. YOLOv11 reduces this issue more effectively than the other models, particularly for comets and globular clusters, while YOLOv8 shows the highest rate of background confusion.

This observation indicates a lack of sufficient negative examples in the training data. A promising solution is to include additional training images that contain only background and no celestial objects. This would allow the models to better learn the visual characteristics of true background regions, potentially improving their ability to distinguish between real objects and background noise. We discuss the construction and integration of such a background-focused dataset in Section 6.6.

### 6.6. Improving Background Awareness via Dataset Augmentation

To quantitatively assess the impact of background augmentation, Table 14 presents a comparison of the mAP@50–95 values for each model before and after incorporating background-only samples. The results demonstrate a consistent improvement across all models, indicating that the introduced augmentation strategy effectively enhances detection performance.

To address the problem of frequent misclassification into the background class, a new augmentation strategy was introduced: for every existing annotated image, one additional background-only image was generated. A custom script was used to automatically extract the largest empty region (i.e., one not containing any annotated objects) from each image, resulting in realistic background samples representative of telescope noise and sky texture. These images were added to the dataset and, following the class balancing procedure described earlier in this section, were split approximately into 79% for training, 11% for validation, and 11% for testing subsets. The slight deviation from the exact 80/10/10 split is due to the dataset balancing across multiple classes.

Table 15 summarizes the final class distribution, including the new background class, which balances the dataset and helps the models better distinguish between true objects and empty regions.

Table 16, Table 17 and Table 18 present the normalized confusion matrices after the background augmentation. The most noticeable improvement is the reduction in false-positive cases where real objects were previously misclassified as background. This effect is particularly prominent for the YOLOv11 model, where galaxy classification improves from 0.66 to 0.69 and comet classification from 0.90 to 0.92. Although marginal, these improvements are consistent and confirm that the additional background samples contributed positively to model performance.

These results suggest that incorporating background-only samples is a simple yet effective strategy to mitigate object misclassifications, especially in astronomy datasets where noise and sky artifacts often resemble faint celestial structures.

### 6.7. Qualitative Results

**Figure 6:**
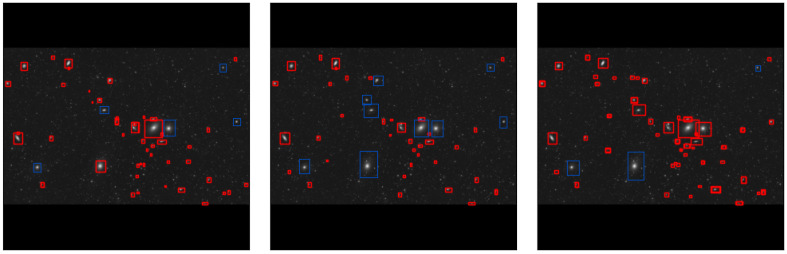
Example detections of galaxies (Markarian’s Chain) from YOLOv8 (**left**), YOLOv9 (**center**), and YOLOv11 (**right**). Missed objects, where no detection was made, are highlighted manually with blue bounding boxes for better illustration.

Figure 6 presents qualitative detection results of each model on sample test images.

From the qualitative results, YOLOv11 demonstrates superior object localization accuracy, particularly in cluttered astronomical images with overlapping objects. YOLOv9 also performs well but exhibits occasional misclassifications. YOLOv8, while accurate, occasionally misses faint objects. Some missed objects were manually annotated with blue bounding boxes to clearly indicate the detection failures.

The experimental results presented at the beginning of Section 6 demonstrate that YOLOv11 achieves superior performance across accuracy, computational efficiency, and generalization ability among the YOLO models. This section provides an in-depth analysis of model performance, discussing key findings, limitations, and potential improvements.

### 6.8. Performance Analysis

The results indicate that YOLOv11 achieves the highest mAP@50–95 (0.6187) and recall (0.8193), while also maintaining the best inference speed (18.21 FPS). This suggests that YOLOv11’s hybrid CNN–Transformer backbone and adaptive receptive field modules contribute to improved object localization, particularly in challenging astronomical images with overlapping objects.

YOLOv9 exhibits a balance between accuracy and efficiency, with mAP@50–95 of 0.6051 and an inference speed of 16.45 FPS. The integration of Transformer-based attention layers and an enhanced Feature Pyramid Network (FPN) allows it to capture fine details more effectively than YOLOv8.

Although YOLOv8 has the highest parameter count (43.6M), its performance does not significantly surpass YOLOv9 or YOLOv11, while requiring more computational resources (164.8 GFLOPs). This suggests that architectural optimizations in YOLOv9 and YOLOv11 enable better accuracy with lower computational costs.

### 6.9. Training Stability and Convergence

The loss curves presented in Figure 5 demonstrate that all three models converge stably, with YOLOv11 reaching the lowest final loss values. Early stopping was triggered at different epochs: YOLOv8: 199 epochs, YOLOv9: 304 epochs, and YOLOv11: 214 epochs.

This indicates that YOLOv9 required the most epochs to reach convergence, suggesting a slower but more stable optimization process. YOLOv8, despite converging faster, shows higher final loss values, indicating potential overfitting or underfitting in some cases.

## 7. Limitations and Challenges

Despite the promising results, several challenges were encountered during the study. One significant limitation was the class imbalance within the dataset, where galaxies were over-represented (3489 images) compared with globular clusters (958 images). This discrepancy may have introduced biases in the models, favoring the detection of more frequent classes. Another challenge was the difficulty in detecting small or faint astronomical objects, especially those embedded in high background noise. This issue affected the accuracy of certain models when processing low-contrast or low-brightness regions.

Another limitation is that the COCO dataset, used for initial pretraining, consists primarily of natural and man-made objects, and its feature priors are not well aligned with the morphology and contrast characteristics typical of astronomical images. More extensive domain-specific fine-tuning, such as pretraining on large-scale astronomical surveys (e.g., Pan-STARRS, SDSS) or using synthetic telescope imagery, could potentially improve the model’s ability to detect faint, small, or highly irregular celestial structures. Additionally, incorporating contrast-aware normalization techniques and custom loss functions sensitive to low signal-to-noise ratios may further enhance detection performance in this specialized context. These enhancements are planned for future work.

To address these limitations in future work, potential improvements include the implementation of advanced dataset augmentation strategies, the use of adaptive loss functions tailored to small object detection, and the adoption of multi-GPU training environments to accommodate more sophisticated model designs.

It is also important to emphasize that all experiments were conducted as part of a master’s thesis project and executed on a single consumer-grade NVIDIA RTX 3060 GPU. This hardware constraint significantly influenced the selection of training hyperparameters, such as batch size, maximum epoch count, and model resolution. In particular, the need to avoid out-of-memory errors and maintain feasible training times restricted the exploration of more computationally demanding architectures and tuning strategies. Consequently, models like YOLOv8, which have higher parameter counts and memory requirements, may not have reached their full performance potential due to these limitations.

## 8. Conclusions

This paper presented a novel dataset for astronomical object detection, COSMICA, along with a comprehensive comparative analysis of several object detection models, YOLOv8, YOLOv9, YOLOv11, EfficientDet-Lite0, and MobileNetV3-FasterRCNN-FPN. Leveraging this dataset, which consists of 5145 manually annotated telescope images, the models were trained, evaluated, and compared based on their accuracy, inference speed, and computational efficiency.

The experimental results demonstrate that YOLOv11 achieves the highest overall detection accuracy (mAP@50–95: 0.6187) with an inference speed of 18.21 FPS and a moderate computational cost (25.28 M parameters, 86.6 GFLOPs), making it the most suitable model for real-time astronomical applications. YOLOv9 provides a balance between accuracy and computational cost, while YOLOv8, despite its larger parameter count, does not significantly outperform the other models and requires higher computational resources. The training and validation loss curves indicate that all three models exhibit stable convergence, with YOLOv11 reaching lower final loss values. However, challenges such as class imbalance and the detection of faint celestial objects remain unresolved.

Our experiments with EfficientDet-Lite0 and MobileNetV3-FasterRCNN-FPN reinforced the importance of high-capacity architectures in astronomical image analysis. Despite EfficientDet-Lite0 and MobileNetV3-FasterRCNN-FPN efficiency, these models failed to detect a wide range of objects reliably, especially under background noise and faint object conditions, achieving significantly lower mAP scores than YOLO models.

This study demonstrated that deep learning models such as YOLOv11 can serve as effective tools for the automatic detection of celestial objects in large-scale sky surveys. The trade-offs between accuracy and computational efficiency should be carefully considered based on the specific application. For real-time applications, such as telescope guidance systems, YOLOv11 is particularly advantageous due to its high accuracy and relatively low computational cost. For large-scale archival analysis, such as processing past astronomical data, YOLOv9 provides a suitable balance between efficiency and precision. In resource-limited environments, such as embedded systems for space telescopes, additional model compression techniques may be necessary to deploy these models effectively. Future research may focus on several directions. Hybrid architectures that combine convolutional neural networks with Transformer-based modules could improve feature extraction and object localization. Model optimization techniques, such as quantization and pruning, may enable deployment on resource-constrained devices like the Raspberry Pi. Additionally, augmenting the dataset with synthetic images could enhance the detection of faint and small objects, while multi-GPU or distributed training could facilitate the use of more advanced models. 

## Figures and Tables

**Table 1:** Dataset class distribution.

Class	Number of Images
Comets	1941
Galaxies	3489
Nebulae	2471
Globular Clusters	958
**Total**	**8859**

**Table 2:** Dataset split distribution across training, validation, and test sets for each class.

Class	Total	Train	Test	Validation
Comets	7832	6147	815	870
Galaxies	16,257	12,844	1620	1793
Globular Clusters	4875	3854	499	522
Nebulae	6654	5231	703	720
**Total**	**35,618**	**28,076**	**3637**	**3905**

**Table 3:** Comparison of YOLO model architectures.

Model	Layers	Parameters	GFLOPs
YOLOv8	268	43.6M	164.8
YOLOv9	384	25.3M	102.3
YOLOv11	464	25.2M	86.6

**Table 4:** Architectural comparison of EfficientDet-Lite0 and MobileNetV3-FasterRCNN-FPN.

Model	Layers	Parameters	GFLOPs
EfficientDet-Lite0	207	3.9M	0.39
MobileNetV3-FRCNN	298	6.2M	1.26

**Table 5:** Training configuration and computational resources.

Parameter	Value
Image Size	640 × 640
Batch Size	16
Epochs	400 (early stopping at 20)
Optimizer	Auto
Learning Rate (lr0)	0.01
Momentum	0.937
Weight Decay	0.0005
IoU Threshold	0.7
Hardware	NVIDIA RTX 3060, AMD Ryzen 7 5800X, 32 GB RAM
Framework	PyTorch 2.0

**Table 6:** Performance comparison of YOLO models on the test set. Best results for each metric are highlighted in bold.

Model	mAP@50–95	mAP@50	Precision	Recall	FPS
YOLOv8	0.5883	0.8729	0.8957	0.8060	14.37
YOLOv9	0.6051	0.8812	0.9013	0.8127	16.45
YOLOv11	**0.6187**	**0.8895**	**0.9082**	**0.8193**	**18.21**

**Table 7:** Evaluation metrics for EfficientDet-Lite0 and MobileNetV3-FasterRCNN-FPN using TorchMetrics.

Model	mAP@50–95	mAP@50	Precision	Recall
EfficientDet-Lite0	0.0827	0.2312	0.153	0.173
MobileNetV3-FRCNN	0.1889	0.4206	0.248	0.3379

**Table 8:** Bootstrapped evaluation metrics (mean and 95% confidence intervals) for each YOLO model.

Metric	Mean	CI
YOLOv8 mAP@50–95	0.6160	[0.6142, 0.6178]
YOLOv8 mAP@50	0.8680	[0.8669, 0.8691]
YOLOv8 Precision	0.9137	[0.9129, 0.9145]
YOLOv8 Recall	0.8102	[0.8095, 0.8109]
YOLOv9 mAP@50–95	0.6449	[0.6432, 0.6466]
YOLOv9 mAP@50	0.8692	[0.8682, 0.8702]
YOLOv9 Precision	**0.9248**	[0.9238, 0.9258]
YOLOv9 Recall	0.8150	[0.8142, 0.8158]
YOLOv11 mAP@50–95	**0.6465**	[0.6449, 0.6481]
YOLOv11 mAP@50	**0.8941**	[0.8924, 0.8958]
YOLOv11 Precision	0.9225	[0.9213, 0.9237]
YOLOv11 Recall	**0.8212**	[0.8199, 0.8225]

**Table 9:** Per-class mAP@50 results. Best results for each class are highlighted in bold.

Model	Comets	Galaxies	Nebulae	Globular Clusters
YOLOv8	0.8512	0.8923	0.8745	0.8698
YOLOv9	0.8627	0.9015	0.8821	0.8756
YOLOv11	**0.8735**	**0.9152**	**0.8914**	**0.8843**

**Table 10:** Per-class mAP@50–95 results. Best results for each class are highlighted in bold.

Model	Comets	Galaxies	Nebulae	Globular Clusters
YOLOv8	0.621	0.472	0.676	0.698
YOLOv9	0.625	0.455	0.665	0.714
YOLOv11	**0.638**	**0.501**	**0.693**	**0.724**

**Table 11:** Normalized confusion matrix for YOLOv8. Rows represent predicted labels; columns represent true labels.

Predicted\True	Comet	Galaxy	Globular Cluster	Nebula	Background
**Comet**	0.89	0.00	0.00	0.00	0.10
**Galaxy**	0.00	0.66	0.02	0.00	0.71
**Globular Cluster**	0.00	0.00	0.94	0.00	0.05
**Nebula**	0.00	0.00	0.00	0.77	0.13
**Background**	0.10	0.33	0.06	0.21	–

**Table 12:** Normalized confusion matrix for YOLOv9. Rows represent predicted labels; columns represent true labels.

Predicted\True	Comet	Galaxy	Globular Cluster	Nebula	Background
**Comet**	0.91	0.00	0.00	0.00	0.11
**Galaxy**	0.00	0.66	0.01	0.00	0.60
**Globular Cluster**	0.00	0.00	0.95	0.00	0.09
**Nebula**	0.00	0.00	0.00	0.75	0.20
**Background**	0.09	0.33	0.05	0.24	–

**Table 13:** Normalized confusion matrix for YOLOv11. Rows represent predicted labels; columns represent true labels.

Predicted\True	Comet	Galaxy	Globular Cluster	Nebula	Background
**Comet**	0.88	0.00	0.00	0.00	0.18
**Galaxy**	0.00	0.59	0.03	0.00	0.64
**Globular Cluster**	0.00	0.00	0.91	0.00	0.10
**Nebula**	0.00	0.00	0.00	0.78	0.09
**Background**	0.12	0.41	0.09	0.19	–

**Table 14:** Comparison of mAP@50–95 values before and after background augmentation.

Model	Before Augmentation	After Background Augmentation
YOLOv8	0.5883	0.5961
YOLOv9	0.6051	0.6147
YOLOv11	**0.6187**	**0.6273**

**Table 15:** Final dataset distribution across training, validation, and test sets, including the background class.

Class	Total	Train	Test	Validation
Comets	7832	6255	781	796
Galaxies	16,263	13,069	1570	1624
Globular Clusters	5014	3983	501	530
Nebulae	12,061	9650	1179	1232
Background	34,903	27,826	3510	3567
**Total**	**76,073**	**60,783**	**7541**	**7749**

**Table 16:** Normalized confusion matrix for YOLOv8 before and after background augmentation. “Before” refers to the model trained without background images, while “After” refers to the model trained with additional background-only images. Each cell shows the value in the format before/after.

Predicted/True	Comet	Galaxy	Globular Cluster	Nebula	Background
**Comet**	0.89/0.92	0.00/0.00	0.00/0.00	0.00/0.00	0.10/0.17
**Galaxy**	0.00/0.00	0.66/0.69	0.02/0.01	0.00/0.00	0.71/0.56
**Globular Cluster**	0.00/0.00	0.00/0.00	0.94/0.97	0.00/0.00	0.05/0.04
**Nebula**	0.00/0.01	0.00/0.00	0.00/0.00	0.77/0.88	0.13/0.22
**Background**	0.10/0.08	0.33/0.30	0.06/0.03	0.21/0.10	–

**Table 17:** Normalized confusion matrix for YOLOv9 before and after background augmentation. “Before” refers to the model trained without background images, while “After” refers to the model trained with additional background-only images. Each cell shows the value in the format before/after.

Predicted/True	Comet	Galaxy	Globular Cluster	Nebula	Background
**Comet**	0.91/0.90	0.00/0.00	0.00/0.00	0.00/0.00	0.11/0.20
**Galaxy**	0.00/0.00	0.66/0.66	0.01/0.02	0.00/0.00	0.60/0.53
**Globular Cluster**	0.00/0.00	0.00/0.00	0.95/0.97	0.00/0.00	0.09/0.07
**Nebula**	0.00/0.01	0.00/0.00	0.00/0.00	0.75/0.86	0.20/0.20
**Background**	0.09/0.10	0.33/0.34	0.05/0.03	0.24/0.12	–

**Table 18:** Normalized confusion matrix for YOLOv11 before and after background augmentation. “Before” refers to the model trained without background images, while “After” refers to the model trained with additional background-only images. Each cell shows the value in the format before/after.

Predicted/True	Comet	Galaxy	Globular Cluster	Nebula	Background
**Comet**	0.88/0.90	0.00/0.00	0.00/0.00	0.00/0.00	0.18/0.19
**Galaxy**	0.00/0.00	0.59/0.66	0.03/0.01	0.00/0.00	0.64/0.55
**Globular Cluster**	0.00/0.00	0.00/0.00	0.91/0.96	0.00/0.00	0.10/0.06
**Nebula**	0.00/0.01	0.00/0.00	0.00/0.00	0.78/0.87	0.09/0.20
**Background**	0.12/0.10	0.41/0.33	0.09/0.04	0.19/0.12	–

## Data Availability

The dataset used in this study is publicly available. The dataset can be accessed at https://www.kaggle.com/datasets/piratinskii/astronomical-object-detection (accessed on 4 May 2025) and https://app.roboflow.com/test-kbeim/astronomical-object-detection/ (accessed on 4 May 2025).

## Abbreviations

The following abbreviations are used in this manuscript:

YOLO	You Only Look Once
CNN	convolutional neural network
mAP	mean Average Precision
GFLOPs	Giga Floating Point Operations per Second
COCO	Common Objects in Context Dataset
